# Hidden Biodiversity in an Ecologically Important Freshwater Amphipod: Differences in Genetic Structure between Two Cryptic Species

**DOI:** 10.1371/journal.pone.0069576

**Published:** 2013-08-13

**Authors:** Anja Marie Westram, Jukka Jokela, Irene Keller

**Affiliations:** 1 Animal and Plant Sciences, University of Sheffield, Sheffield, United Kingdom; 2 Department of Biology, Eidgenössische Technische Hochschule Zürich, Zürich, Switzerland; 3 Aquatic Ecology, Eawag (Swiss Federal Institute of Aquatic Science and Technology), Dübendorf, Switzerland; 4 Fish Ecology and Evolution, Eawag (Swiss Federal Institute of Aquatic Science and Technology) Kastanienbaum, Switzerland; 5 Department of Aquatic Ecology and Macroevolution, University of Bern, Bern, Switzerland; Onderstepoort Veterinary Institute, South Africa

## Abstract

Cryptic species, i.e. species that are morphologically hard to distinguish, have been detected repeatedly in various taxa and ecosystems. In order to evaluate the importance of this finding, we have to know in how far cryptic species differ in various aspects of their biology. The amphipod *Gammarus fossarum* is a key invertebrate in freshwater streams and contains several cryptic species. We examined the population genetic structure, genetic diversity and demographic history of two of them (type A and type B) using microsatellite markers and asked whether they show significant differences. We present results of population genetic analyses based on a total of 37 populations from the headwaters of two major European drainages, Rhine and Rhone. We found that, in both species, genetic diversity was geographically structured among and within drainages. For type A in the Rhine and type B in the Rhone, we detected significant patterns of isolation by distance. The increase of genetic differentiation with geographical distance, however, was much higher in type A than in type B. This result indicates substantial interspecific differences in population history and/or the extent of current gene flow between populations. In the Rhine, type B does not show evidence of isolation by distance, and population differentiation is relatively low across hundreds of kilometres. The majority of these populations also show signatures of recent bottlenecks. These patterns are consistent with a recent expansion of type B into the Rhine drainage. In summary, our results suggest considerable and previously unrecognized interspecific differences in the genetic structure of these cryptic keystone species.

## Introduction

A comprehensive understanding of biodiversity requires knowledge about the species forming an ecosystem as well as the genetic diversity within these species. Identifying species may not be straightforward, e.g. when they are morphologically hardly distinguishable “cryptic species” [1]. More and more molecular studies reveal that cryptic species are common and not restricted to certain taxonomic groups or ecosystems [2]. Several studies found divergence between cryptic species in ecological, physiological or behavioural aspects [1], [3], [4]. This suggests that the identification of cryptic species and differences between them may be essential for understanding population dynamics and ecosystem functioning.

Multiple cryptic species complexes have been identified in freshwater vertebrates (e.g. [5], [6]) as well as invertebrates (e.g. [7], [8]). Several ecologically important “species” have thus been shown to consist of several reproductively isolated entities (e.g. [9]), the biological differences between which are often unknown.

Amphipoda is an order of mostly aquatic crustaceans in which cryptic species have been found many times. Several amphipods play a central role in freshwater ecosystems as key shredders of decomposing material, important prey items for predators (e.g. fish, [10], [11]), and intermediate hosts for parasites [12]. Multiple studies have shown high levels of population differentiation [13] as well as the evolution of reproductively isolated cryptic species [14]–[16] within morphological species. The fact that such pronounced genetic structure arises despite frequently observed large population sizes with hundreds to thousands of individuals per m^2^ [17]–[19] suggests substantial limitations of gene flow between populations. On the other hand, there are multiple rapidly spreading invasive amphipods (e.g. [20], [21]), suggesting that at least in some species rapid dispersal across large geographical scales is possible.

Because of these discrepancies and their ecological importance, amphipods represent particularly interesting study objects to analyse intraspecific patterns of gene flow and divergence as well as interspecific differences in these patterns. Here, we study both aspects using the cryptic *Gammarus fossarum* species complex and ask in how far cryptic species differ with respect to population genetic structure.

The amount and distribution of genetic diversity within a species reflects both current and historical factors that influence rates of gene exchange between populations [22]. Two main extrinsic factors are likely to shape the population genetic structure of temperate stream species like *G. fossarum*: the association of dispersal with the spatial structure of the river systems, and the climatic history of Pleistocene glaciations. In line with the first prediction, several studies have recorded that genetic divergence increases with waterway distance between populations in many stream species [23], [24]. Also an increasing number of molecular studies shows that multiple species are subdivided into genetic clusters associated with drainage basins [25]–[29].

Pleistocene glaciations wiped out many temperate species from Central Europe and confined them to glacial refugia mostly in southern Europe [30]. After the retreat of the ice, Central Europe was recolonized from these refugia. Multiple species still show a reduction of genetic diversity in the direction of recolonization, representing a signature of repeated bottlenecks during the colonization process ([31] and references therein).

*G. fossarum* is common in smaller streams in prealpine areas and mainly occurs in upstream areas [32], leading to potentially highly isolated populations. *G. fossarum* may occur in extremely high population densities, frequently representing the most abundant macroinvertebrate in streams of our study area (own observations). However, *G. fossarum* may be threatened by extinction locally, for example due to pollution [33]. Previous research has shown that *G. fossarum* represents at least three cryptic species [14], [34], [35], which are probably several million years old and reproductively isolated [14], but morphologically not clearly distinct [36]. In Central Europe, the two most commonly detected species (type A and type B) differ in their geographical distribution, with an eastern type A and a western type B, but their distribution ranges overlap in a contact zone within the Rhine drainage in Germany and Switzerland [14], [35], [37]. This distribution pattern has been interpreted as a legacy of the Pleistocene glaciations, during which type A and B are thought to have persisted in separate refugia (south-east and south-west, respectively), from where they recolonized Central Europe after the retreat of the ice [14], [35].

Several population genetic studies have been conducted on *G. fossarum*, but often focused on limited geographical regions containing only one of the cryptic species and/or were constrained by the limited availability of highly variable and selectively neutral markers [13], [14], [35], [38]–[40]. We here make use of nine recently developed microsatellite markers [41] to examine the intraspecific genetic structure within two cryptic species and potential differences between them. Specifically, we analyse (i) the effect of geographical distance and drainage boundaries on population genetic structure, (ii) the presence of distinct genetic clusters within species, (iii) the effects of recolonization history.

## Methods

### Sampling and genotyping

**Figure 1:**
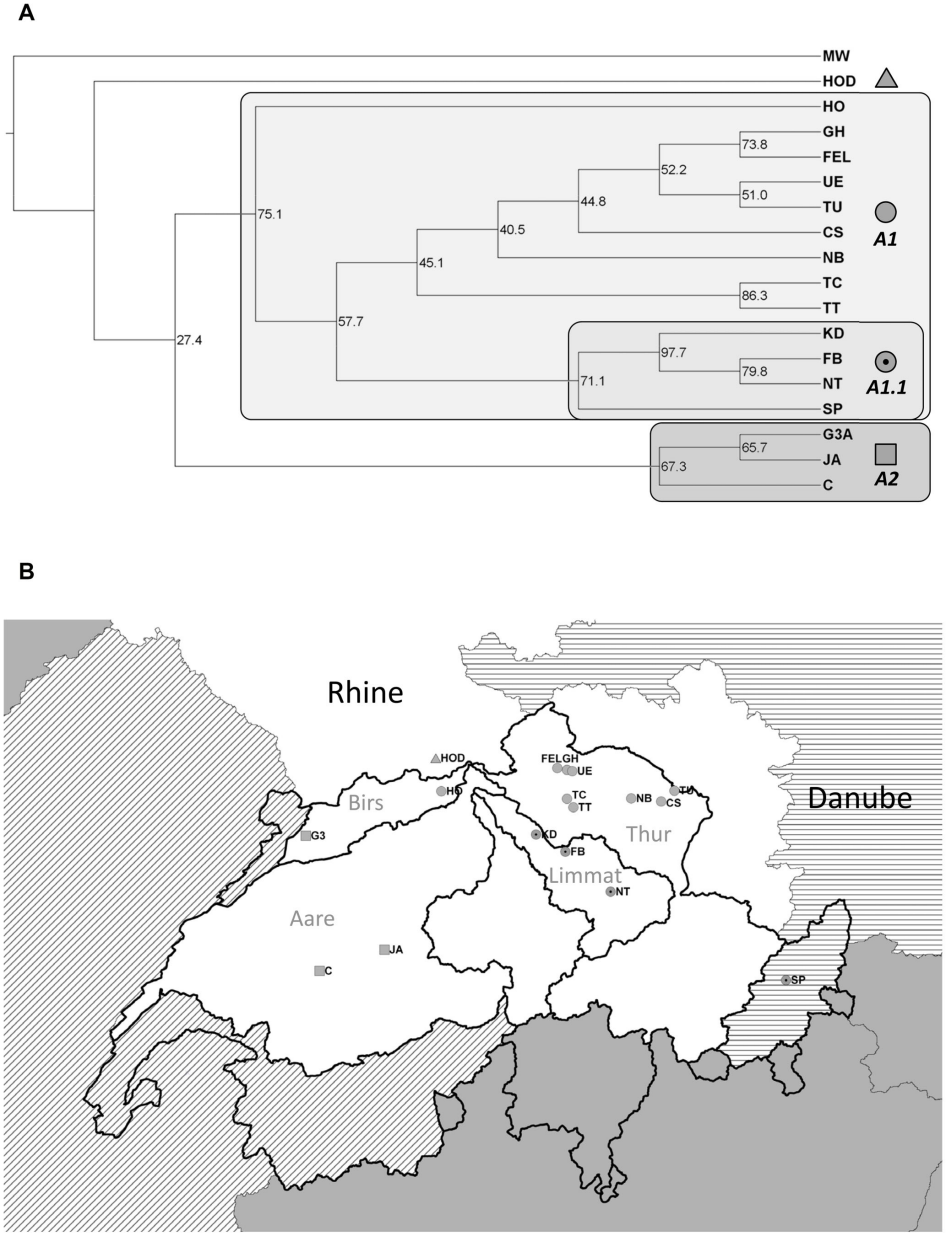
Population tree and geographical distribution of clusters for *G. fossarum* type A (MW: type B/outgroup). The tree (a) was calculated using the Neighbour Joining method; numbers at nodes indicate bootstrap support in % (1000 bootstraps). Main clusters are named and given a symbol. The same symbols are used to indicate the geographical location of the populations in the map (b). The three major European drainages sampled in this study are shown in different shadings. The border of Switzerland and delimitations of Swiss subdrainages are indicated by thick black lines. Relevant subdrainages of the Rhine in Switzerland are labelled (grey). None of the populations showed evidence of recent bottlenecks.

**Figure 2:**
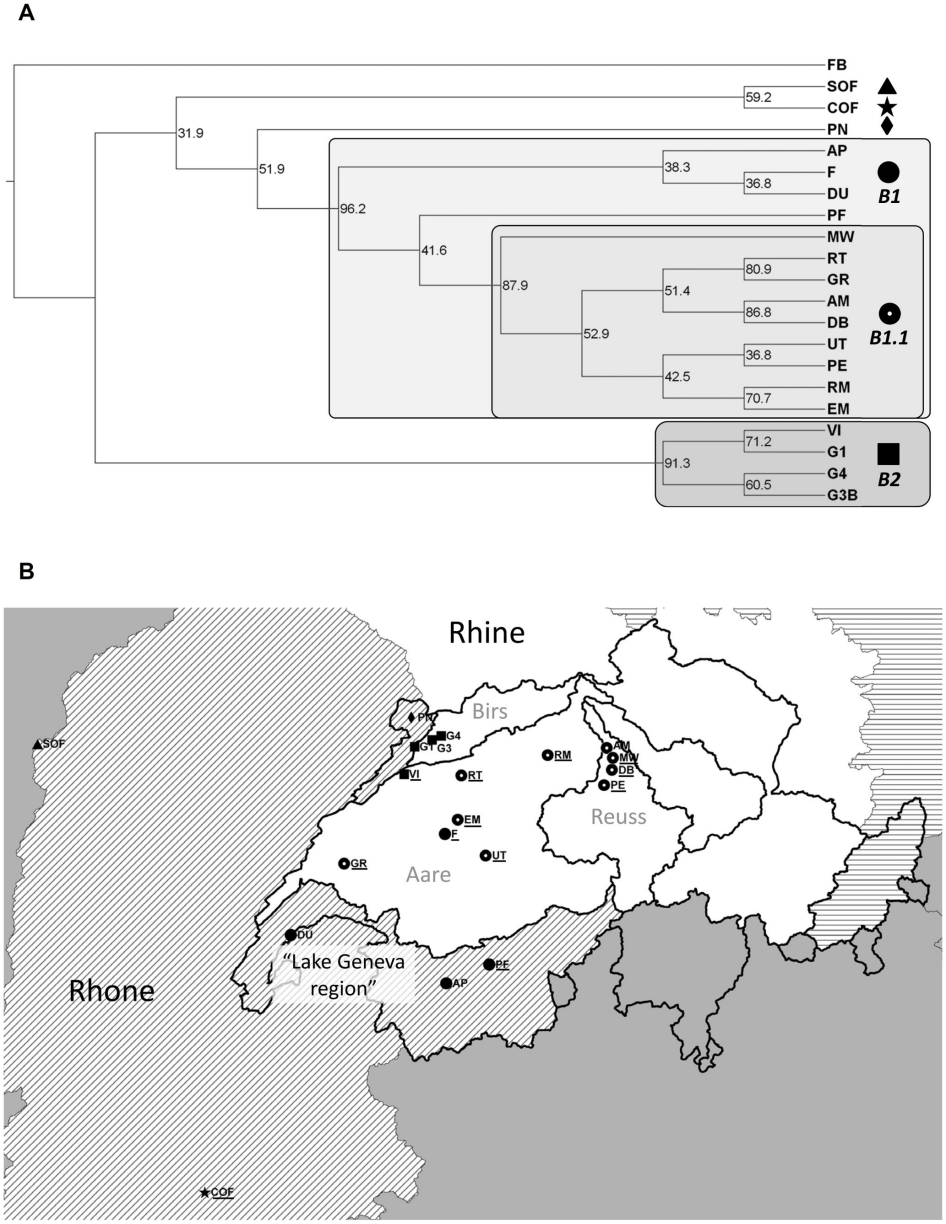
Population tree and geographical distribution of clusters for *G. fossarum* type B (FB: type A/outgroup). The tree (a) was calculated using the Neighbour Joining method; numbers at nodes indicate bootstrap support in % (1000 bootstraps). Main clusters are named and given a symbol. The same symbols are used to indicate the geographical location of the populations in the map (b). The three major European drainages sampled in this study are shown in different shadings. The border of Switzerland and delimitations of Swiss subdrainages are indicated by thick black lines. Relevant subdrainages of the Rhine in Switzerland are labelled (grey). The area referred to as “Lake Geneva region” in the text is also indicated. Names of populations showing evidence for recent bottlenecks (as indicated by the program BOTTLENECK using the infinite alleles mutation model) are underlined.

We sampled *G. fossarum* from 36 sites (“populations”) within three major European drainages (Rhine, Rhone and Danube; Fig. 1 and Fig. 2; Table S1 in Supporting Information) for most of which molecular species identification based on 16S pyrosequencing had been conducted in a previous study [37]. Sixteen type A and 14 type B samples were from the Rhine drainage, which contains the contact zone between the two species. In the Rhone drainage, where type A has not been found, we sampled 6 type B populations. One type A population was sampled from the Danube drainage. All sampling sites were located in Switzerland except one type A population from the Rhine drainage (HOD, southern Germany) and the two westernmost Rhone populations (COF and SOF, France). Only one site (G3) contained both cryptic species. Individuals were collected by kick-sampling all available microhabitats and subsequently stored in 70% ethanol. DNA was extracted from either complete animals or heads [42].

We amplified nine polymorphic, unlinked microsatellite loci (gf08, gf 13, gf18, gf19, gf21, gf22, gf24, gf27, gf28) specifically designed for both cryptic species in 14 to 35 individuals per population following the protocol of Westram *et al.* [41]. The amplified fragments were diluted and mixed with GeneScan LIZ500 size standard. Subsequently, they were run on a 3730×l DNA Analyzer (ABI), and scored in the program GeneMarker 1.8. At several loci, we occasionally detected individuals with more than two alleles. As other studies confirm that *G. fossarum* are diploid (Westram *et al.* unpublished data; Drees *et al.* personal communication), it seems likely that these patterns reflect contamination during DNA extraction. Gammarids may be cannibalistic, they carry their eggs and young in a brood pouch, and we typically stored multiple individuals in the same tube, which could all have led to the occasional co-extraction of DNA from non-target individuals. We excluded these individuals, as well as those with unexpected peak size patterns (e.g. smaller peak for the shorter allele) from the analysis (ca. 10% of all individuals).

In type B, locus gf27 was monomorphic, and locus gf21 showed evidence of null alleles (see below) and contained alleles which could not be reliably scored. We therefore excluded gf21 and gf27 from analyses of type B. These loci were also excluded from the type A data set when a direct comparison of results between species was desirable. However, F_ST_ values calculated with and without these loci, respectively, were very similar (data not shown), indicating no major bias caused by the inclusion/exclusion of these loci. One locus (gf28) could not be scored in population G3A as peaks were irregular in size and shape. This population was excluded from analyses requiring data for all loci.

### Basic population genetic statistics

Using the program Fstat (version 2.9.3.2), we tested all loci and sampling sites for F_IS_ values (6660 randomizations) significantly deviating from Hardy-Weinberg equilibrium.

Genetic distances between pairs of sampling sites were calculated as pairwise F_ST_ [43] in Fstat. Significance was assessed based on 13320 permutations. As F_ST_ is not independent of marker variability, we additionally calculated the newer measure D_est_ [44] using the online application SMOGD [45] with 1000 randomizations.

As a measure of genetic diversity, we calculated allelic richness per population based on 13 diploid individuals (the lowest sample size available) using rarefaction and averaged over loci in the program hp-rare [46]. We tested for interspecific differences in allelic richness (Mann-Whitney U test) and its variance (Levene's test of equality of error variances) in SPSS.

### Effects of watersheds and geographical distances on population genetic structure

We first asked whether geographically close sampling sites also cluster genetically. Because genetic divergence within species is large (see Results section), methods based on Hardy-Weinberg equilibrium, e.g. Structure [47], are not appropriate. We therefore constructed Neighbour Joining population trees using programs from the PHYLIP package. Note that we use the trees only for identification of higher-level clusters based on genetic similarities, and do not make phylogenetic inferences. We used distance matrices (Cavalli-Sforza chord distances) to create trees separately for the two species (each analysis including one population of the respective other species as an outgroup). Extended majority rule consensus trees were created from 1000 bootstrap trees.

To assess the relevance of the clusters identified in the trees, we used analysis of molecular variance (AMOVA; [48]), which partitions the genetic variance between different hierarchical levels (in this case: within populations, between populations within clusters, and between clusters).

We tested for isolation by distance (IBD) within each species and drainage (Rhine/Rhone) using the program IBDWS [49]. Genetic distance was measured as F_ST_/(1-F_ST_) [50]. Two different measures of geographical distance were tested. First, we used the straight line distance between pairs of populations. Second, we calculated the shortest pairwise distance along waterways (Swisstopo 1∶25000 vector map, 2007, for Rhine samples; River and Catchment Database for Europe (CCM), 2003, for Rhone samples) in ESRI ArcMap 9.2.

The slope of the IBD relationship indicates how strongly genetic divergence increases with geographical distance. To test for interspecific differences in this respect, we analysed whether the 95% confidence intervals of the slopes overlapped between the two species.

### Testing for the presence of genetically diverged clusters within species

We identified highly divergent clusters in the phylogenetic trees for both species (see Results section). We hypothesized that gene flow might be reduced between these genetic clusters, on top of the effect of geographical distance alone. To test for that, we performed partial Mantel tests in Fstat, including an indicator matrix specifying whether two populations were in the same (0) or different genetic clusters (1). These tests were only performed for type A and B in the Rhine.

### Testing for indications of historical differences between the species

Recolonization from glacial refugia is thought to have proceeded from east to west in type A and from west to east in type B [14], [35]. We tested for a reduction of allelic richness in the proposed direction of colonization in each species. Preliminary analyses revealed large subdrainage effects on allelic richness, especially in type A. Therefore we did not perform a regression analysis but compared allelic richness between different groups of populations (i.e. subdrainages). For type A, we tested for a significant difference between the central (Limmat) and eastern (Thur) populations using a Mann-Whitney U test. We did not make statistical comparisons involving the western (Aare) populations or the remaining three type A populations, as fewer than three populations had been sampled per (sub)drainage. For type B, we tested for differences among three geographic regions (from west to east: French/Jurassic (Rhone), Lake Geneva region (southern Swiss Rhone populations), and Rhine) using ANOVA followed by Tamhane's T2 multiple comparisons test, which does not assume equality of variances, in SPSS.

Recent changes in effective population size leave signatures in the allele frequency distribution in the population. Specifically, bottlenecks cause a loss of rare alleles, leading to a heterozygosity excess compared to the expectation at mutation-drift equilibrium, while population expansions cause heterozygosity deficiency [51]. We tested for deviations of the expected Hardy-Weinberg heterozygosity from the expected equilibrium heterozygosity using the program BOTTLENECK [52]. Our markers almost certainly do not conform to a strict stepwise mutation model, and the best mutation model was not clear *a priori*. We therefore ran analyses with all possible mutation models (infinite alleles model/IAM, stepwise mutation model/SMM, and two-phase model/TPM, which represents a combination of the former two) with 1000 replications each. We applied the Wilcoxon test (one-sided) implemented in BOTTLENECK as this test is powerful if the number of loci is small, and appropriate for low sample sizes [52].

### Ethics statement

No specific permits were required for the described field studies. In Switzerland, France and Germany, work with Gammarus does not require permission and waterbodies are not private property if nothing else is indicated. Samples were not taken from streams where private property was indicated or from nature reserves. The field studies did not involve endangered or protected species.

## Results

### Basic population genetic statistics

Locus gf21 showed elevated F_IS_ values in eight out of 20 type B populations (P-values<0.05), indicating the presence of null alleles. Otherwise, no locus showed consistently elevated F_IS_ values across populations. For the French type B population SOF, we detected significantly elevated F_IS_ values for six loci, so results for this population should be interpreted with caution. Other populations did not show deviations from Hardy-Weinberg equilibrium across multiple loci, suggesting the absence of within-population genetic substructure. .

Although type B was studied on a larger geographical scale than type A (maximum straight line distance within A: 230 km; within B: 300 km) and included two major drainages, overall F_ST_ was higher in type A (0.38; 95% CI: 0.302–0.475) than type B (0.19; 95% CI: 0.125–0.267). This difference remained when we used the alternative measure D_est_ (A: 0.51 vs. B: 0.28; averaged over loci), demonstrating that the F_ST_ differences are not caused by interspecific differences in marker variability (see Table S2 and Table S3 in Supporting Information for pairwise F_ST_ and D_est_ values). Most pairwise F_ST_ values were significantly different from 0 (132 out of 136 in type A; 170 out of 190 in type B). In type A, all non-significant comparisons were between geographically close populations within the Thur subdrainage of the Rhine. In type B, only comparisons within the same genetic cluster (see below; Fig. 2) generated non-significant F_ST_ values.

### Effects of watersheds and geographic distance on population genetic structure

The consensus tree for type A contained two major clusters with bootstrap support of 75% (A1) and 67% (A2) (Fig. 1). The clusters coincided with geography: Cluster A1 contained all eastern populations from the Rhine drainage as well as the Danubian population. Within this eastern cluster, a smaller cluster (cluster A1.1; bootstrap 71%) contained three adjacent populations from the same subdrainage (Limmat) and the Danubian population. Cluster A2 contained the three more western Rhine populations (C and JA, Aare subdrainage; G3A, Birs subdrainage). The northernmost population HOD was not included in any of the main clusters.

In type B, we also detected two main geographically sorted clusters (Fig. 2). All Rhine populations except the north-western ones formed one large cluster (B1; bootstrap 96%) together with the Rhone populations from southern Switzerland (“Lake Geneva region”). Within this cluster, a subcluster (B1.1; bootstrap 88%) contained all Rhine populations except population F. The second main cluster (B2; bootstrap 91%) contained four north-western Rhine drainage populations (G1, G3B and G4, Birs subdrainage; VI, Aare subdrainage). The French and Jura populations (SOF, COF and PN; Rhone) were not placed in any of the main clusters.

The AMOVA results showed that in both type A and type B the identified clusters explained a substantial part of genetic variation (type A: 28.1%; type B: 23.0%). In type A the variation observed between populations within clusters (16.7%) was larger than in type B (5.1%; all p<0.0001).

**Figure 3:**
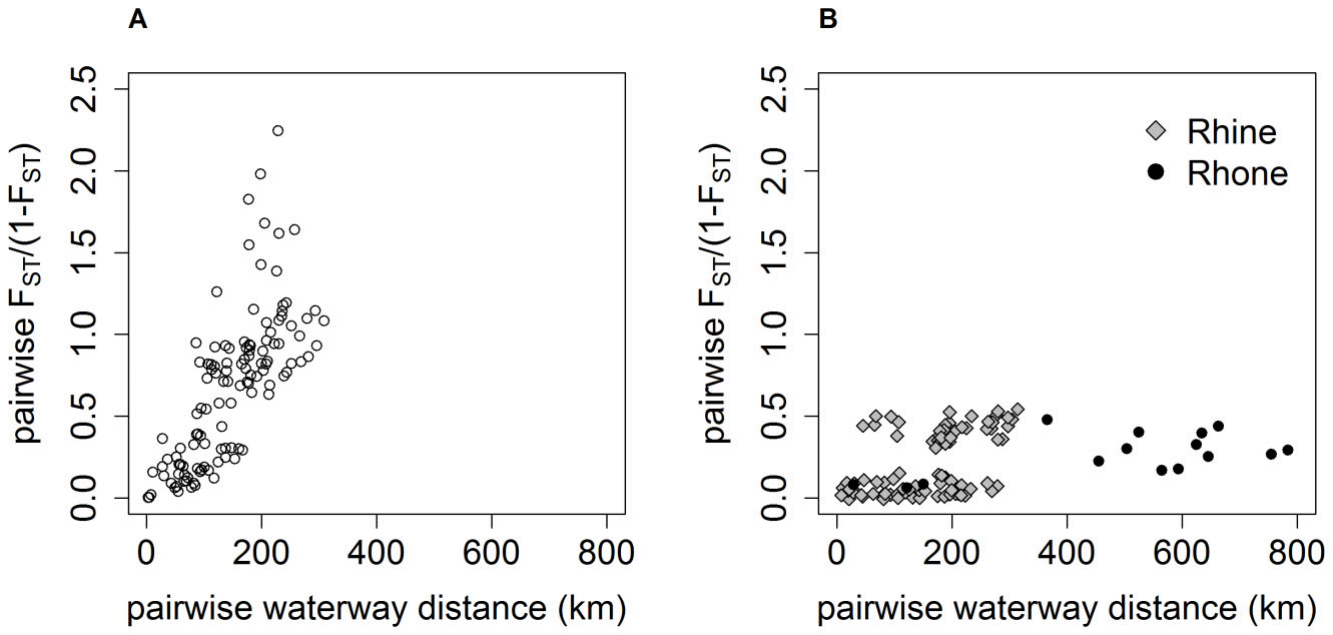
Isolation by distance plots for *G. fossarum* type A (left) and type B (right). Only data for the Rhine drainage are shown for type A. For type B, data for the Rhine (grey diamonds) and Rhone (black circles) are shown. The relationship was significant in Mantel tests for type A (P = 0.0001) as well as type B in the Rhone (P = 0.0191), but not for type B in the Rhine (Table 1). Slopes are significantly different between species as indicated by non-overlapping 95% confidence intervals (Table 1). For type B in the Rhine drainage, the group with a higher degree of differentiation (F_ST_/(1-F_ST_)>0.3) contains pairs of populations from the two distinct clusters B1 and B2 (Fig. 2).

For the type A populations from the Rhine, we found evidence of IBD using Mantel tests (Fig. 3; Table 1). The genetic distance showed a significant increase with both measures of geographic distance, with waterway distance being a better predictor of genetic distance than straight line distance (Table 1). IBD was still evident when testing only within cluster A1 (excluding A1.1; tests were not run for A1.1 and A2 due to small sample sizes; see also Figure S1).

**Table 1:** Results of Mantel tests conducted to test for isolation by distance (geographical distance vs. F_ST_/(1-F_ST_)) in the program IBDWS for the cryptic *Gammarus fossarum* species type A and B in different drainages.

group^1^	distance type	Mantel r	p^2^	IBD slope^3^	95% CI^3^
A (Rhine)	waterway	0.7382	0.0001***	0.0063	0.0045–0.0081
A (Rhine)	straight line	0.6316	0.0003***	0.0102	0.0073–0.0131
A (cluster A1)	waterway	0.6632	0.0013**	0.0023	0.0016–0.0031
A (cluster A1)	straight line	0.4811	0.0356*	0.0031	0.0005–0.0058
B (cluster B1 Rhine)	waterway	0.0510	0.3282	-	-
B (cluster B1 Rhine)	straight line	0.0358	0.269	-	-
B (Rhone)	waterway	0.5832	0.0191*	0.0006	−0.0001–0.0012
B (Rhone)	straight line	0.7305	0.023*	0.002	0.0006–0.0035

1 “group” indicates the species and drainage included in the analysis. Two main clusters of type B were observed in the Rhine (see Fig. 2). Isolation by distance was assessed only within the larger cluster, B1.

2 ***P<0.001; **P<0.01; *P<0.05.

3 The regression slopes (IBD slope) and their confidence intervals (95% CI) are indicated if significantly different from zero. The slopes were significantly higher in type A (Rhine) than type B (Rhone) irrespective of the distance measure used.

The type B populations from the Rhone drainage also showed significant evidence of IBD (Fig. 3; Table 1). Here, straight line distance was a better predictor than waterway distance (Table 1). In contrast, type B populations from the Rhine did not show a pattern of IBD (Fig. 3).

We compared the slopes of the significant IBD relationships between type A (Rhine) and type B (Rhone). Independent of the distance measure used, the non-overlapping 95% confidence intervals indicated that the slopes were significantly different between the two species (Table 1, see also Fig. 3). For waterway distance, this difference was still evident when only cluster A1 of type A was included.

### Distinct genetic clusters within species

A partial Mantel test showed that genetic clusters in type A explain a significant proportion of the genetic differentiation between populations, on top of the effect of geographical distance alone (Table 2). For type B in the Rhine, genetic cluster also had a significant effect on population differentiation, while geographical distance was not significant (Table 2).

**Table 2:** Results of partial Mantel test, investigating the effect of clusters (A1.1, other A1, A2 for type A; B1, B2 for type B) on genetic differentiation when correcting for geographical distance.

group	variable	Mantel r	p^1^
A (Rhine)	waterway distance	0.76	0.0013**
A (Rhine)	cluster	0.31	0.0009***
B (Rhine)	waterway distance	0.48	0.7266
B (Rhine)	cluster	0.84	0.0001***

1 ***P<0.001; **P<0.01.

### Indications of historical differences between the species

While the mean allelic richness did not differ between species (MW U-test, U = 159, P = 0.975), the range was larger in type A than in type B (Table S1 in Supporting Information; range A, 2.63–5.34; range B, 2.76–4.44; Levene's test for equality of variances, F_1,34_ = 18.1, P<0.001).

**Figure 4:**
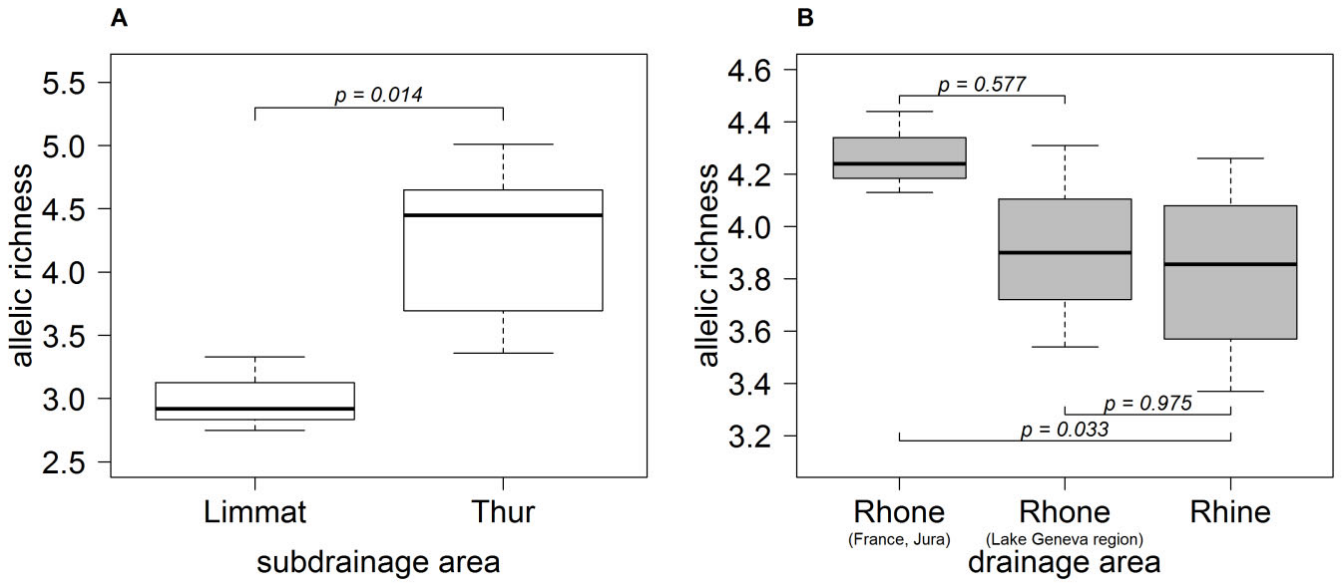
Allelic richness (average across loci) for different geographical groups of *Gammarus fossarum*. a) Difference between geographical groups of type A populations (Thur: east; Limmat: central). See Fig. 1 for location of subdrainages. The P-value (Mann-Whitney U test) is indicated. b) Difference among three geographical groups of type B populations. See Fig. 2 for location of geographical regions. “Rhone (France, Jura)” includes all Rhone populations except those in the Lake Geneva region. “Rhine” includes all Rhine type B populations except the genetically distinct cluster B2 (see Figure 2). P-values (Tamhane's T2 multiple comparisons test) are indicated.

We found that allelic richness varied between different geographical groups of populations. In type A, we found significantly higher allelic richness in the eastern (Thur) than in the central subdrainage (Limmat) (MW U-test U = 0, P = 0.014; Fig. 4). The western populations C and JA had low values similar to those in the Limmat subdrainage (Table S1 in Supporting Information), but were not tested due to the limited number of samples (n = 2).

In type B, we found that allelic richness was significantly higher in the French/Jurassic Rhone populations than in the Rhine (Fig. 4, Tamhane's T2, P = 0.033), while the Lake Geneva region did not differ from the other two regions (Fig. 4; cluster B2 excluded).

The detection of heterozygosity or homozygosity excess strongly depended on the mutation model we used. We report only overall patterns here (for details see Table S1 in Supporting Information). Type A populations never showed evidence of heterozygosity excess (i.e., population bottlenecks). However, under the TPM (SMM), 3 (6) populations showed evidence of heterozygosity deficiency, 2 (3) of which were populations from the Limmat (i.e. central) subdrainage.

Under the IAM, all but one cluster B1.1 populations showed evidence of population bottlenecks. This number however was reduced to 1 (0) under the TPM (SMM). Other type B populations from cluster B1 (i.e., excluding B1.1) and from the Rhone did not show a clear pattern of heterozygosity excess or deficiency.

## Discussion

We show that two morphologically cryptic species show similar population genetic patterns reflecting stream topology and postglacial colonization history. However, the two species differ substantially with regard to the extent of genetic differentiation between populations: While type B is characterized by moderate genetic differentiation across hundreds of kilometres, even geographically close populations are markedly distinct in type A, suggesting differences in the time since colonization and/or the extent of intraspecific gene flow. Our results demonstrate that genetic data may reveal differences hidden by morphological stasis and, in line with other studies, support the importance of considering cryptic species in basic research and conservation.

### Effects of watersheds and geographical distances on population genetic structure

In both species, the population genetic structure is shaped by drainage boundaries. In type A, we find that populations from the same Rhine subdrainages generally cluster together in the tree (except populations G3 and HO; Fig. 1). The populations from the Limmat subdrainage, for example, form a separate cluster within the large eastern Swiss cluster. In type B, the Birs subdrainage contains a cluster that is genetically very different from the clusters in other subdrainages. Similar drainage-specific lineages have been found in other stream species, particularly fish [25]–[29].

Some specific patterns, however, indicate that dispersal is not strictly limited to waterways. Cluster B2, for example, contains all type B populations from the Birs subdrainage, but also an Aare population (VI), which is geographically close, but distant along waterways. Similarly, the type A population from the Danube drainage (SP) clusters with Rhine populations (cluster A1.1), and the three Lake Geneva region populations cluster with most of the Rhine populations (cluster B1; Fig. 2). A previous study on *G. fossarum* type A using allozyme markers detected a similar pattern on a smaller geographical scale, where genetic divergence between closely adjacent Rhine and Danube populations was not considerably larger than that observed within the Danube drainage [13]. Such patterns suggest recent or on-going gene flow over land, which is not surprising as aquatic species, including *Gammarus*, may be transported by waterfowl [53]–[56] and humans [57], [58]. Additionally, flooding might produce temporary connections between usually unconnected streams. Watersheds might therefore present less of a barrier to gene flow than observed in other purely aquatic organisms (e.g. [59]).

Underlining the finding that geographically proximate populations are also genetically more similar, we detected a significant IBD along waterways within two groups of populations: type A populations in the Rhine, and type B populations in the Rhone (Fig. 3). This pattern indicates that gene flow is more likely between neighbouring than distant populations, as would be expected in species with limited dispersal along streams.

### Distinct genetic clusters within species

Genetic differentiation between *G. fossarum* populations (within types) is often remarkably high, with some F_ST_ values reaching the level of interspecific comparisons in other taxa. Similarly, earlier allozyme studies found large genetic differences between geographically close *G. fossarum* type A populations [13], [38], and Alp *et al.* [40] showed that populations within the same stream system separated by less than 20 km can be genetically clearly distinct. These findings are probably related to the fact that *G. fossarum* is typically restricted to the upper reaches of streams, leading to a very patchy distribution. This may also explain why the degree of genetic structure was found to be higher in *G. fossarum* type A than in other *Gammarus* species with a more continuous distribution in the lower reaches of streams [38].

The pronounced restrictions to dispersal might reduce gene flow between distant sites to a minimum. However, our partial Mantel tests indicate that the distinct clusters detected in type A are genetically even more differentiated than expected based on geographical distance alone. This may be explained by the fact that geographically distant streams are usually connected by large rivers, which present a barrier to *G. fossarum* dispersal, and may include additional obstacles like waterfalls and anthropogenic barriers.

In type B, we also find evidence for two strongly isolated groups within a cryptic species (clusters B1 and B2). B2 might represent a lineage of type B with a different geographical origin (e.g. from more northern parts of the Rhine drainage). Interestingly, the 16S mtDNA haplotypes of individuals from population VI (cluster B2) belong to a clade widespread in the north (Germany), while all other mtDNA haplotypes we obtained from Switzerland (from B1 populations) had not been observed in Germany ([14]; Westram *et al.*, unpublished data). Secondary contact between two distinct type B lineages, one from the north (B2) and one from the southwest/Rhone drainage (B1) is therefore plausible. Based on the high degree of differentiation and the fact that there is no evidence for intermediate populations, reproductive isolation between these clusters is an interesting possibility that deserves further investigation.

### Indications of historical and biological differences between the species

Founder events lead to a loss of genetic diversity [60], [61]. Therefore, during colonization processes, a reduction of genetic diversity in the direction of colonization is expected, and has often been detected for species that colonized Europe after the Pleistocene glaciations ([31] and references therein). In accordance with the hypothesis that type A recolonized Europe from the east, we found that allelic richness is much higher in the eastern than in the central and western populations (Fig. 4; Table S1). Likewise, in accordance with the hypothesis that type B expanded from the Rhone into the Lake Geneva region and then the Rhine drainage, we found that allelic richness is highest in the French and Jurassic Rhone populations (Fig. 4). Our data therefore suggest that signatures of postglacial recolonization are still detectable, and support previous hypotheses about the direction of colonization. It is worth noting that such a pattern implies that colonization involved the crossing of drainage boundaries; e.g., if colonization had been strictly limited to waterways, all Rhine drainage sites would have been colonized from the northern, downstream regions of the Rhine drainage, which would not produce a longitudinal cline in allelic richness.

One of our most interesting findings is that the extent of genetic differentiation between populations is much higher in type A than type B on similar geographical scales. This is reflected by two different tests performed in this study. First, AMOVA indicates that, in type A, a larger proportion of the total genetic variation is explained by differences between clusters and populations. Second, while both type A (Rhine) and type B (Rhone) show isolation by distance, the slope of the relationship is markedly steeper in type A than type B (Fig. 3; Table 1). This difference persists when only cluster A1 of type A is included in the test in order to avoid potentially confounding effects of the large differentiation between clusters (see above).

Systematic differences between the east and the west of our study area could theoretically contribute to the observed pattern, as species and geographical area are not completely independent in this comparison. For example, the east could be characterized by more dispersal barriers, leading to more isolated *Gammarus* populations and, consequently, a higher degree of population differentiation. However, in the Rhine drainage, where both species coexist, type B also shows much lower levels of differentiation between populations. Lower F_ST_ values in type B than type A were also found in a small-scale study within the Sense subdrainage of the Rhine, where the two species occur in close spatial proximity (Alp *et al.*, unpublished data). These findings indicate that the differences in differentiation levels are species differences instead of geographical effects.

**Figure 5:**
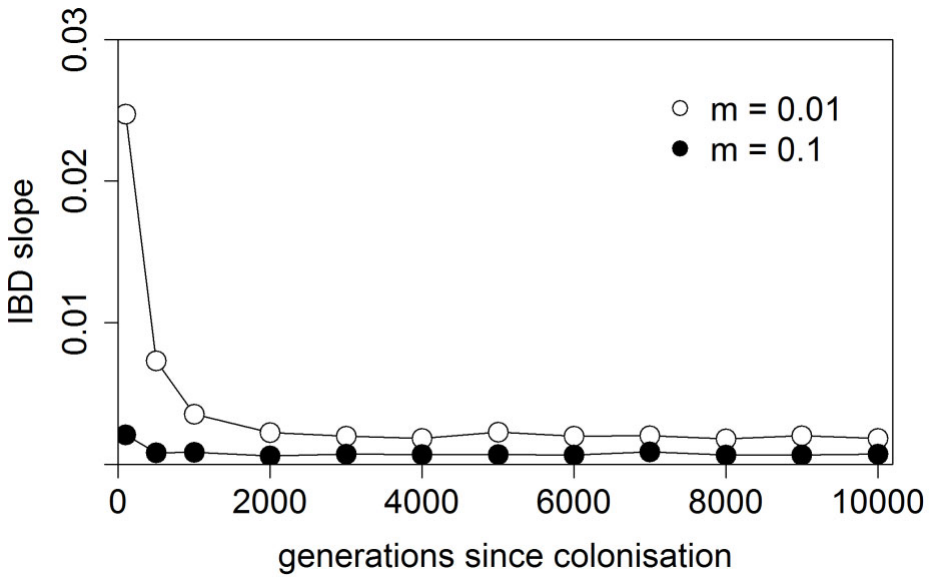
Changes in the slope of the isolation-by-distance relationship following a linear stepping-stone colonization process. Data were simulated in the program QuantiNemo (Neuenschwander *et al.*, 2008) for 50 demes where, initially, only one edge deme was occupied. Colonization and migration took place only between neighbouring demes at a rate m (colonization rate and migration rate are equal). The carrying capacity of each deme was N = 1000, and newly established demes grew instantly to carrying capacity. We simulated 9 loci with a maximum of 7 alleles per locus, assuming a K-allele mutation model and a mutation rate of μ = 5*10^−4^. Initial allele frequencies were generated in the program Easypop (Balloux, 2001) to match the average number of alleles observed in our real data set. The recolonization process was completed within the first 50–70 generations. IBD was significant directly after colonization (after 100 generations) as well as at migration-drift equilibrium (after 10 000 generations). The graph shows that the observed steeper slope in type A could be explained by a lower migration rate and/or more recent colonization of the area.

Such differences could reflect interspecific differences in the number of migrants per generation, i.e. in migration rate and/or effective population size [62]. Alternatively, recent demographic processes might explain the observed pattern. Specifically, differences in the time since colonization of the studied area can substantially affect genetic differentiation between populations [63], [64]. To visualize the effects of different factors (migration rate, time since colonization) on patterns of isolation by distance (IBD), we simulated a stepwise colonization process in a linear habitat (Fig. 5; see figure legend for simulation details). Significant IBD was found shortly (ca. 30–50 generations) after all habitat patches had been colonized, as well as at migration-drift equilibrium (i.e. when population divergence had stabilized). As Fig. 5 shows, the slope of the IBD relationship is substantially affected both by differences in the time since colonization and migration rates, and peaks directly after colonization. The same is true for average population differentiation, which can be high shortly after colonization due to repeated founder events, but then decreases as a result of the homogenizing effect of gene flow [63], [64].

To explain the observed interspecific differences in population structure purely as differences in the time since colonisation, we would have to postulate that colonization by type A was more recent than by type B, and involved strong founder events (see Fig. 5). Consequently, we would expect stronger signals of potential population bottlenecks in type A than B while, in fact, we observe the opposite. We therefore suggest that factors other than a recent colonisation by type A are likely to produce the distinct patterns between the two species. Specifically, the higher between-population F_ST_s and steeper IBD slope in type A would be consistent with lower N_e_ and/or m compared to type B. Accordingly, other studies have shown that closely related species may differ in the extent of gene flow between populations, e.g. due to differences in dispersal ability [65], life history [66]) or population sizes [67]. While we cannot clearly distinguish between different possible explanations with the current data set, further work focussing on biological differences between the species could address them in more detail.

Type B in the Rhine does not show evidence of IBD, but signatures of recent bottlenecks are detectable in a large number of populations. One possible explanation for this is a relatively recent colonization of this area by type B. Bottlenecks in the early stages of colonization followed by a rapid spread across the drainage might explain why IBD cannot be observed.

## Conclusion

As earlier studies have shown, amphipods may be characterized by strongly isolated populations, but on the other hand some species may rapidly spread across large geographical regions. We show that markedly different patterns of population genetic structure can even be observed between relatively closely related cryptic species.

The observed interspecific differences have important implications. First, the lower degree of population differentiation in type B might reflect a higher degree of connectivity between populations, which might reduce extinction risk in a metapopulation [68], [69]. For type A, the extinction of a single population will represent a comparatively greater loss of genetic diversity. On the other hand, the potential for local adaptation in a given population may be higher [70].

Cryptic species are most likely present within many endangered taxa and keystone taxa of ecosystems. Two findings of this study, the presence of isolated clusters within both cryptic species and the interspecific differences in the degree of population differentiation, call for detailed investigations of cryptic species. This study underlines the importance of using molecular markers not only to identify them, but also to characterize their population genetic structure to detect other “cryptic” patterns which may be of ecological and evolutionary importance.

## Supporting Information

Figure S1 **Isolation-by-distance plot for** ***Gammarus fossarum*** **type A.** Each dot (population pair) is coloured based on the genetic cluster the two compared populations belong to (see Fig. 1 of manuscript; “A1” in the legend refers to A1 populations which are not included in the sub-cluster A1.1). Filled symbols = within-cluster comparisons; empty symbols = between-cluster comparisons. The relationship was significant for the within-cluster comparison A1 - A1 (other within-cluster comparisons not tested).
(TIFF)

Table S1 ***Gammarus fossarum*** **sampling sites, allelic richness and results of bottleneck tests.**
(DOC)

Table S2 **F_ST_ (above diagonal) and D_est_ (below diagonal) values for** ***Gammarus fossarum*** **type A population pairs.**
(DOC)

Table S3 **F_ST_ (above diagonal) and D_est_ (below diagonal) values for** ***Gammarus fossarum*** **type B population pairs.**
(DOC)
