# Supplementary figures and images for: Hidden Biodiversity in an Ecologically Important Freshwater Amphipod: Differences in Genetic Structure between Two Cryptic Species
Source: PLoS One. 2013 Aug 13;8(8):e69576. doi: 10.1371/journal.pone.0069576 (PMC3742660; doi:10.1371/journal.pone.0069576)

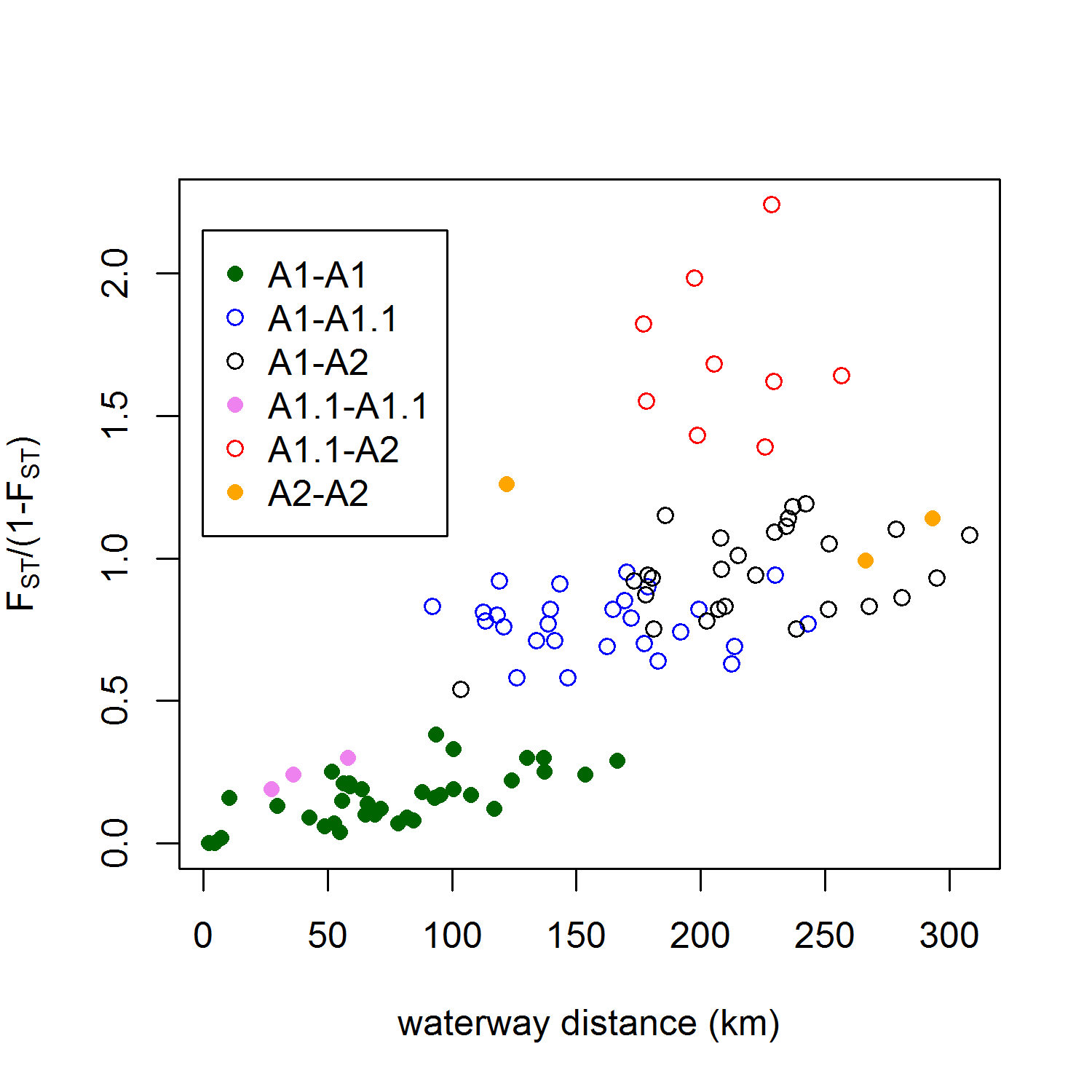

Supplement: Figure S1 — Isolation-by-distance plot for Gammarus fossarum type A. Each dot (population pair) is coloured based on the genetic cluster the two compared populations belong to (see Fig. 1 of manuscript; “A1” in the legend refers to A1 populations which are not included in the sub-cluster A1.1). Filled symbols = within-cluster comparisons; empty symbols = between-cluster comparisons. The relationship was significant for the within-cluster comparison A1 - A1 (other within-cluster comparisons not tested). (TIFF) [file pone.0069576.s001.tiff]
