# Supplementary material for: Hidden Biodiversity in an Ecologically Important Freshwater Amphipod: Differences in Genetic Structure between Two Cryptic Species
Source: PLoS One. 2013 Aug 13;8(8):e69576. doi: 10.1371/journal.pone.0069576 (PMC3742660; doi:10.1371/journal.pone.0069576)
Supplement: Table S1 — Gammarus fossarum sampling sites, allelic richness and results of bottleneck tests. (DOC) [file pone.0069576.s002.doc]

|  |  |  |  |  |  |  |  | **bottleneck P^5^** | | | | | |
| --- | --- | --- | --- | --- | --- | --- | --- | --- | --- | --- | --- | --- | --- |
|  |  |  |  |  |  |  |  | **IAM** | | **TPM** | | **SMM** | |
| **drainage** | **site** | **species** | **AR^1^** | **country^2^** | **latitude^3^** | **longitude^3^** | **N^4^** | **def.** | **exc.** | **def.** | **exc.** | **def.** | **exc.** |
| Danube | SP | A | 3.24 | CH | 46.693 | 10.090 | 35 | 0.96 | 0.06 | 0.95 | 0.15 | 0.29 | 0.77 |
| Rhine | C | A | 2.63 | CH | 46.762 | 7.302 | 22 | 0.47 | 0.59 | 0.23 | 0.81 | 0.15 | 0.95 |
| Rhine | CS | A | 4.41 | CH | 47.444 | 9.370 | 27 | 0.71 | 0.34 | 0.23 | 0.81 | 0.06 | 0.96 |
| Rhine | FB | A | 3.33 | CH | 47.246 | 8.785 | 27 | 0.5 | 0.59 | **0.03** | 0.98 | **0.02** | 1 |
| Rhine | FEL | A | 5.01 | CH | 47.583 | 8.803 | 24 | 0.29 | 0.77 | **0.04** | 0.97 | **<0.01** | 1 |
| Rhine | G3A | A | n.d. | CH | 47.319 | 7.218 | 28 | 0.5 | 0.59 | 0.11 | 0.92 | 0.11 | 0.92 |
| Rhine | GH | A | 4.8 | CH | 47.591 | 8.744 | 20 | 0.77 | 0.29 | 0.15 | 0.95 | **0.03** | 0.98 |
| Rhine | HO | A | 5.09 | CH | 47.501 | 8.038 | 25 | 0.81 | 0.23 | 0.53 | 0.53 | 0.23 | 0.81 |
| Rhine | HOD | A | 5.34 | D | 47.638 | 8.007 | 18 | 0.59 | 0.47 | 0.06 | 0.96 | **0.02** | 0.99 |
| Rhine | JA | A | 2.97 | CH | 46.850 | 7.691 | 24 | 0.81 | 0.23 | 0.47 | 0.59 | 0.06 | 0.96 |
| Rhine | KD | A | 2.92 | CH | 47.319 | 8.609 | 24 | 0.31 | 0.89 | 0.08 | 0.95 | **0.05** | 0.97 |
| Rhine | NB | A | 3.72 | CH | 47.460 | 9.189 | 29 | 0.81 | 0.23 | 0.34 | 0.71 | 0.06 | 0.96 |
| Rhine | NT | A | 2.75 | CH | 47.077 | 9.053 | 26 | 0.06 | 0.97 | **0.03** | 1 | **0.03** | 1 |
| Rhine | TC | A | 3.67 | CH | 47.463 | 8.800 | 18 | 0.53 | 0.53 | 0.19 | 0.85 | 0.06 | 0.96 |
| Rhine | TT | A | 3.36 | CH | 47.428 | 8.838 | 30 | 0.71 | 0.34 | 0.53 | 0.53 | 0.06 | 0.96 |
| Rhine | TU | A | 4.5 | CH | 47.487 | 9.452 | 14 | 0.85 | 0.19 | 0.59 | 0.47 | 0.15 | 0.95 |
| Rhine | UE | A | 4.49 | CH | 47.578 | 8.835 | 18 | 0.53 | 0.53 | 0.29 | 0.77 | 0.19 | 0.85 |
| Rhine | AM | B | 4.21 | CH | 47.280 | 8.382 | 23 | 0.96 | 0.06 | 0.77 | 0.29 | 0.47 | 0.59 |
| Rhine | DB | B | 3.84 | CH | 47.180 | 8.415 | 24 | 0.98 | **0.03** | 0.81 | 0.23 | 0.53 | 0.53 |
| Rhine | EM | B | 4.26 | CH | 46.957 | 7.389 | 20 | 1 | **0.01** | 0.96 | 0.06 | 0.22 | 0.92 |
| Rhine | F | B | 3.37 | CH | 46.892 | 7.304 | 19 | 0.96 | 0.06 | 0.92 | 0.22 | 0.42 | 0.66 |
| Rhine | G1 | B | 2.93 | CH | 47.288 | 7.101 | 32 | 0.89 | 0.31 | 0.5 | 0.59 | 0.11 | 0.92 |
| Rhine | G3B | B | 3.83 | CH | 47.319 | 7.218 | 23 | 0.66 | 0.41 | 0.41 | 0.66 | **0.04** | 0.97 |
| Rhine | G4 | B | 3.83 | CH | 47.337 | 7.279 | 25 | 0.47 | 0.59 | 0.15 | 0.95 | 0.06 | 0.96 |
| Rhine | GR | B | 3.4 | CH | 46.755 | 6.639 | 27 | 0.98 | **0.03** | 0.81 | 0.23 | 0.47 | 0.59 |
| Rhine | MW | B | 3.88 | CH | 47.234 | 8.423 | 29 | 0.97 | **0.04** | 0.95 | 0.15 | 0.47 | 0.59 |
| Rhine | PE | B | 3.68 | CH | 47.111 | 8.362 | 30 | 0.97 | **0.04** | 0.96 | 0.06 | 0.85 | 0.19 |
| Rhine | RM | B | 3.87 | CH | 47.249 | 7.989 | 22 | 0.99 | **0.01** | 0.98 | **0.03** | 0.77 | 0.2 |
| Rhine | RT | B | 3.57 | CH | 47.158 | 7.413 | 23 | 1 | **<0.01** | 0.85 | 0.19 | 0.34 | 0.71 |
| Rhine | UT | B | 4.08 | CH | 46.794 | 7.573 | 27 | 0.99 | **0.02** | 0.66 | 0.411 | 0.59 | 0.47 |
| Rhine | VI | B | 2.76 | CH | 47.163 | 7.032 | 19 | 1 | **0.02** | 0.97 | **0.05** | 0.59 | 0.5 |
| Rhone | AP | B | 3.9 | CH | 46.212 | 7.315 | 34 | 0.95 | 0.15 | 0.77 | 0.29 | 0.29 | 0.77 |
| Rhone | COF | B | 4.24 | F | 45.250 | 5.766 | 21 | 0.98 | **0.02** | 0.78 | 0.28 | 0.08 | 0.95 |
| Rhone | DU | B | 4.31 | CH | 46.427 | 6.294 | 23 | 0.81 | 0.23 | 0.29 | 0.77 | **0.03** | 0.98 |
| Rhone | PF | B | 3.54 | CH | 46.298 | 7.596 | 23 | 0.98 | **0.02** | 0.98 | **0.04** | 0.78 | 0.28 |
| Rhone | PN | B | 4.44 | CH | 47.421 | 7.078 | 22 | 0.53 | 0.53 | 0.41 | 0.66 | 0.15 | 0.95 |
| Rhone | SOF | B | 4.13 | F | 47.265 | 4.587 | 27 | 0.47 | 0.59 | 0.23 | 0.81 | **0.04** | 0.97 |

^1^ AR = allelic richness based on 13 diploid individuals as calculated by the program hp-rare.

^2^ Countries: CH = Switzerland; D = Germany; F = France.

^3^ Locations are indicated using Universal Transverse Mercator (UTM) system coordinates

^4^ N = sample size

^5^ “bottleneck P” indicates the Wilcoxon test P-value (one tailed) calculated by the program BOTTLENECK for heterozygosity deficiency (“def.”) and excess (“exc.”), respectively, for different mutation models (IAM = Infinite Alleles Model, TPM = Two-Phase Model, SMM = Stepwise Mutation model; see main text). Significant P-values are in bold.
