# Supplementary material for: Hidden Biodiversity in an Ecologically Important Freshwater Amphipod: Differences in Genetic Structure between Two Cryptic Species
Source: PLoS One. 2013 Aug 13;8(8):e69576. doi: 10.1371/journal.pone.0069576 (PMC3742660; doi:10.1371/journal.pone.0069576)
Supplement: Table S2 — FST (above diagonal) and Dest (below diagonal) values for Gammarus fossarum type A population pairs. (DOC) [file pone.0069576.s003.doc]

|  | **C** | **CS** | **FB** | **FEL** | **GH** | **HO** | **HOD** | **JA** | **KD** | **NB** | **NT** | **SP** | **TC** | **TT** | **TU** | **UE** |
| --- | --- | --- | --- | --- | --- | --- | --- | --- | --- | --- | --- | --- | --- | --- | --- | --- |
| **C** | - | 0.48 | 0.58 | 0.53 | 0.52 | 0.49 | 0.47 | 0.56 | 0.63 | 0.52 | 0.62 | 0.53 | 0.53 | 0.54 | 0.52 | 0.54 |
| **CS** | 0.63 | - | 0.45 | 0.09 | 0.09 | 0.19 | 0.23 | 0.45 | 0.47 | 0.12 | 0.49 | 0.33 | 0.11 | 0.18 | 0.13 | 0.11 |
| **FB** | 0.66 | 0.58 | - | 0.44 | 0.42 | 0.45 | 0.45 | 0.59 | 0.16 | 0.39 | 0.19 | 0.25 | 0.45 | 0.37 | 0.39 | 0.42 |
| **FEL** | 0.85 | 0.10 | 0.39 | - | 0.00 | 0.14 | 0.28 | 0.45 | 0.45 | 0.07 | 0.46 | 0.29 | 0.17 | 0.16 | 0.08 | 0.00 |
| **GH** | 0.88 | 0.10 | 0.37 | 0.00 | - | 0.15 | 0.25 | 0.44 | 0.44 | 0.06 | 0.45 | 0.29 | 0.20 | 0.17 | 0.06 | 0.02 |
| **HO** | 0.68 | 0.21 | 0.62 | 0.15 | 0.19 | - | 0.27 | 0.43 | 0.45 | 0.23 | 0.48 | 0.34 | 0.27 | 0.25 | 0.23 | 0.15 |
| **HOD** | 0.71 | 0.31 | 0.73 | 0.47 | 0.41 | 0.37 | - | 0.41 | 0.49 | 0.30 | 0.48 | 0.37 | 0.34 | 0.35 | 0.23 | 0.28 |
| **JA** | 0.73 | 0.67 | 0.86 | 0.58 | 0.54 | 0.56 | 0.67 | - | 0.61 | 0.45 | 0.62 | 0.53 | 0.52 | 0.50 | 0.46 | 0.45 |
| **KD** | 0.68 | 0.61 | 0.05 | 0.40 | 0.43 | 0.49 | 0.74 | 0.72 | - | 0.41 | 0.23 | 0.30 | 0.48 | 0.37 | 0.43 | 0.43 |
| **NB** | 0.82 | 0.08 | 0.30 | 0.04 | 0.04 | 0.25 | 0.50 | 0.62 | 0.34 | - | 0.41 | 0.24 | 0.16 | 0.15 | 0.08 | 0.04 |
| **NT** | 0.67 | 0.60 | 0.05 | 0.39 | 0.41 | 0.61 | 0.70 | 0.85 | 0.06 | 0.29 | - | 0.23 | 0.49 | 0.41 | 0.43 | 0.44 |
| **SP** | 0.69 | 0.44 | 0.12 | 0.30 | 0.33 | 0.47 | 0.58 | 0.89 | 0.15 | 0.18 | 0.07 | - | 0.31 | 0.27 | 0.21 | 0.27 |
| **TC** | 0.62 | 0.06 | 0.31 | 0.07 | 0.13 | 0.35 | 0.56 | 0.78 | 0.31 | 0.09 | 0.33 | 0.23 | - | 0.14 | 0.23 | 0.17 |
| **TT** | 0.73 | 0.19 | 0.19 | 0.06 | 0.09 | 0.30 | 0.58 | 0.67 | 0.15 | 0.10 | 0.23 | 0.19 | 0.04 | - | 0.20 | 0.12 |
| **TU** | 0.82 | 0.11 | 0.30 | 0.06 | 0.06 | 0.29 | 0.42 | 0.78 | 0.41 | 0.07 | 0.34 | 0.20 | 0.18 | 0.14 | - | 0.07 |
| **UE** | 0.88 | 0.10 | 0.31 | 0.00 | 0.01 | 0.16 | 0.50 | 0.60 | 0.33 | 0.02 | 0.31 | 0.25 | 0.08 | 0.04 | 0.05 | - |
