# Supplementary material for: Hidden Biodiversity in an Ecologically Important Freshwater Amphipod: Differences in Genetic Structure between Two Cryptic Species
Source: PLoS One. 2013 Aug 13;8(8):e69576. doi: 10.1371/journal.pone.0069576 (PMC3742660; doi:10.1371/journal.pone.0069576)
Supplement: Table S3 — FST (above diagonal) and Dest (below diagonal) values for Gammarus fossarum type B population pairs. (DOC) [file pone.0069576.s004.doc]

|  | **AM** | **AP** | **COF** | **DB** | **DU** | **EM** | **F** | **G1** | **G3B** | **G4** | **GR** | **MW** | **PE** | **PF** | **PN** | **RM** | **RT** | **SOF** | **UT** | **VI** |
| --- | --- | --- | --- | --- | --- | --- | --- | --- | --- | --- | --- | --- | --- | --- | --- | --- | --- | --- | --- | --- |
| **AM** | - | 0.07 | 0.15 | -0.01 | 0.12 | 0.01 | 0.13 | 0.29 | 0.25 | 0.26 | 0.03 | 0.07 | 0.04 | 0.11 | 0.24 | 0.02 | 0.00 | 0.29 | 0.02 | 0.26 |
| **AP** | 0.07 | - | 0.15 | 0.08 | 0.06 | 0.05 | 0.07 | 0.27 | 0.23 | 0.23 | 0.09 | 0.09 | 0.05 | 0.08 | 0.21 | 0.07 | 0.08 | 0.28 | 0.07 | 0.26 |
| **COF** | 0.19 | 0.19 | - | 0.13 | 0.18 | 0.13 | 0.14 | 0.33 | 0.29 | 0.28 | 0.16 | 0.16 | 0.12 | 0.15 | 0.25 | 0.15 | 0.14 | 0.23 | 0.14 | 0.31 |
| **DB** | 0.00 | 0.07 | 0.15 | - | 0.14 | 0.01 | 0.11 | 0.31 | 0.28 | 0.28 | 0.01 | 0.06 | 0.03 | 0.11 | 0.24 | 0.02 | 0.00 | 0.28 | 0.02 | 0.29 |
| **DU** | 0.09 | 0.03 | 0.23 | 0.16 | - | 0.10 | 0.09 | 0.29 | 0.26 | 0.27 | 0.15 | 0.14 | 0.08 | 0.08 | 0.20 | 0.13 | 0.13 | 0.29 | 0.10 | 0.27 |
| **EM** | 0.00 | 0.04 | 0.16 | 0.00 | 0.11 | - | 0.08 | 0.34 | 0.30 | 0.30 | 0.02 | 0.08 | 0.02 | 0.10 | 0.24 | 0.00 | 0.01 | 0.30 | 0.02 | 0.31 |
| **F** | 0.13 | 0.02 | 0.14 | 0.10 | 0.04 | 0.07 | - | 0.35 | 0.32 | 0.32 | 0.09 | 0.12 | 0.09 | 0.12 | 0.25 | 0.13 | 0.10 | 0.25 | 0.09 | 0.33 |
| **G1** | 0.21 | 0.18 | 0.33 | 0.22 | 0.19 | 0.23 | 0.22 | - | 0.04 | 0.08 | 0.35 | 0.31 | 0.29 | 0.30 | 0.36 | 0.34 | 0.33 | 0.35 | 0.30 | 0.07 |
| **G3B** | 0.20 | 0.13 | 0.30 | 0.20 | 0.19 | 0.21 | 0.22 | 0.02 | - | 0.02 | 0.32 | 0.25 | 0.26 | 0.27 | 0.30 | 0.29 | 0.30 | 0.28 | 0.26 | 0.04 |
| **G4** | 0.27 | 0.20 | 0.33 | 0.28 | 0.27 | 0.28 | 0.31 | 0.03 | 0.01 | - | 0.33 | 0.24 | 0.25 | 0.26 | 0.29 | 0.29 | 0.30 | 0.30 | 0.26 | 0.08 |
| **GR** | 0.01 | 0.08 | 0.16 | 0.00 | 0.16 | 0.01 | 0.07 | 0.22 | 0.25 | 0.34 | - | 0.07 | 0.05 | 0.13 | 0.29 | 0.05 | -0.01 | 0.30 | 0.03 | 0.33 |
| **MW** | 0.05 | 0.09 | 0.17 | 0.04 | 0.12 | 0.06 | 0.10 | 0.23 | 0.16 | 0.16 | 0.03 | - | 0.05 | 0.10 | 0.26 | 0.10 | 0.07 | 0.29 | 0.05 | 0.27 |
| **PE** | 0.03 | 0.03 | 0.13 | 0.02 | 0.07 | 0.00 | 0.07 | 0.15 | 0.13 | 0.19 | 0.04 | 0.04 | - | 0.06 | 0.24 | 0.05 | 0.04 | 0.30 | 0.02 | 0.27 |
| **PF** | 0.11 | 0.05 | 0.18 | 0.09 | 0.08 | 0.07 | 0.10 | 0.17 | 0.14 | 0.17 | 0.10 | 0.09 | 0.03 | - | 0.23 | 0.13 | 0.11 | 0.30 | 0.09 | 0.29 |
| **PN** | 0.26 | 0.19 | 0.28 | 0.26 | 0.16 | 0.27 | 0.19 | 0.32 | 0.26 | 0.31 | 0.28 | 0.26 | 0.22 | 0.21 | - | 0.26 | 0.27 | 0.32 | 0.24 | 0.30 |
| **RM** | 0.01 | 0.06 | 0.20 | 0.01 | 0.14 | 0.00 | 0.13 | 0.29 | 0.24 | 0.28 | 0.05 | 0.09 | 0.02 | 0.08 | 0.33 | - | 0.02 | 0.29 | 0.05 | 0.32 |
| **RT** | 0.00 | 0.08 | 0.16 | 0.00 | 0.14 | 0.00 | 0.09 | 0.23 | 0.23 | 0.31 | 0.00 | 0.03 | 0.03 | 0.09 | 0.28 | 0.02 | - | 0.29 | 0.03 | 0.31 |
| **SOF** | 0.38 | 0.27 | 0.19 | 0.32 | 0.23 | 0.34 | 0.13 | 0.31 | 0.25 | 0.32 | 0.30 | 0.28 | 0.31 | 0.28 | 0.33 | 0.38 | 0.32 | - | 0.27 | 0.31 |
| **UT** | 0.02 | 0.05 | 0.16 | 0.01 | 0.08 | 0.00 | 0.07 | 0.22 | 0.19 | 0.26 | 0.02 | 0.04 | 0.01 | 0.08 | 0.22 | 0.04 | 0.02 | 0.32 | - | 0.27 |
| **VI** | 0.23 | 0.18 | 0.34 | 0.24 | 0.24 | 0.25 | 0.23 | 0.02 | 0.01 | 0.04 | 0.27 | 0.23 | 0.19 | 0.22 | 0.29 | 0.35 | 0.26 | 0.27 | 0.25 | - |
